# Quantitative electroencephalographic changes and hippocampal atrophy in diabetic patients with mild cognitive impairment in Ismailia region

**DOI:** 10.1186/s41983-018-0018-y

**Published:** 2018-06-01

**Authors:** Ahmed Abo hagar, Yossri Ashour, Reda Abd El-Razek, Mohamed Elsamahy, Osama Shehab

**Affiliations:** 1 Department of Neuropsychiatry, Suez Canal University, Ismailia, Egypt; 2 Department of Neurology, Faculty of Medicine, Suez Canal University, Ismailia, Egypt

**Keywords:** Diabetes mellitus, Mild cognitive impairment, Hippocampal atrophy, QEEG

## Abstract

**Background:**

Cognitive decline could start or get worse among elderly patients with diabetes mellitus more than elderly without diabetes mellitus. So, those diabetic elderly patients have more risk to develop Alzheimer’s disease and vascular dementia.

**Patients and Methods:**

This study included 48 elderly, grouped into three equal groups. First group included patients with diabetes mellitus and cognitive impairment. Second group included patients with diabetes mellitus and no cognitive impairment. The last group included the controls. Evaluation through Mini Mental State Examination, MRI brain, and Quantitative Electroencephalography (QEEG) recording was done for every studied elderly.

**Results:**

MRI finding revealed that hippocampal atrophy was significantly more prevalent among diabetic patients with mild cognitive impairment (MCI) (37.5%). The QEEG showed increase in the distribution of alpha 1 (low alpha) waves among control and diabetic patients without MCI groups, while there was an increase in the distribution of alpha 2 (high alpha) among diabetic patients with MCI. The QEEG results revealed increased alpha 2/alpha 1 ratio among patients with hippocampal atrophy.

**Conclusions:**

Type 2 DM was suggested to increase the risk of cognitive impairment. The cognitive impairment in patients with diabetes mellitus was associated with changes in hippocampal volume and QEEG changes.

## Background

Diabetes mellitus is a common disease known to have adverse effects on all systems of the body (Kodl and Seaquist 2008)**.** Cognitive decline could start or get worse among elderly patients with diabetes mellitus more than elderly without diabetes mellitus. So, those diabetic elderly patients have a higher risk of developing Alzheimer’s disease and vascular dementia (Das et al. 2007)**.** Mild cognitive impairment is a stage between normal cognitive changes with aging and very early dementia (Petersen and Negash 2008)**.** Attention and memory formation take place in the hippocampus. The hippocampus is the main source of rhythmic activity in EEG (Tsanov et al. 2011)**.** It is widely accepted that the cerebral EEG rhythms reflect underlying brain network activity. So, the modifications in these rhythms could be an early sign of Alzheimer’s disease. Specifically, the study of alpha rhythm could be a satisfying measurement for the relationship between the structure and the function of these brain networks (Ingber and Nunez 2011).

### Aim of the work

The aim of this work was to assess the effect of diabetes mellitus on cognitive functions, through assessing quantitative EEG changes and hippocampal atrophy in diabetic patients with mild cognitive impairment.

### Patients and methods

This is a case-control study, including 32 diabetic patients with variable levels of education with at least 8 years of education selected by the attendants in the neuropsychiatry outpatient clinic in Suez Canal University Hospital, Ismailia, Egypt. Patients were subdivided into one group of patients with diabetes mellitus and cognitive impairment and a second group which included patients with diabetes mellitus and no cognitive impairment. The inclusion criteria were the following: controlled diabetic patients with type 2 diabetes, patient aged 55–85 years old, complaint by the patient or report by a relative of memory or other cognitive disturbances, patients with minimal or no depression (according to Beck Depression Inventory) (Beck et al. 1996), and the Arabic translated form of Mini-Mental State Examination (MMSE) with score of 24–27/30 (American Psychiatric Association (APA) 2013)***.***

The exclusion criteria were the following: illiterate individuals or less than 8 years of education, any patient with disturbed conscious level, history or neurological signs of vascular or degenerative disorder, other psychiatric diseases, dementia (MMSE score below 24), epilepsy, patient with moderate or severe depression (according to Beck Depression Inventory), and using psychoactive drugs which included acetylcholinesterase inhibitors and included drugs that enhance brain cognitive functions or bias EEG activity. Also, the systemic diseases that could affect cognitive function and the history or current intake of alcohol or drug addiction were excluded.

Another 16 age-, sex-, and education-matched healthy subjects were included as a control group. The study was approved by the Suez Canal Faculty of Medicine Ethical Committee. Written, informed consent was obtained from all persons before inclusion in the study.

## Methods

All subjects underwent the following:

A. Clinical assessment including thorough history taking, full general, and neurological examination.
B. Routine laboratory investigations: fasting, post-prandial blood sugar level and HBA1C, lipid profile, liver, kidney and thyroid function tests, complete blood count, ESR, uric acid, and Na, K and calcium levels. These tests were done for exclusion of any systemic diseases.
C. Clinical diagnosis of mild cognitive impairment (MCI) according to DSM-V criteria (American Psychiatric Association (APA) 2013).
D. Cognitive assessment using the Mini Mental State Examination (MMSE) (Folstein et al. 1975).

### Measurement of the hippocampus

Coronal and sagittal magnetic radiographic volumetric scan with high-resolution T1 weight intensity was done. The Philips Acheiva scanner 1.5 T, Netherlands (Holland), was used. The used technique was the gradient echo 3D with repetition (TR) of 20 ms and echo (TE) of 5 ms. The used Flip Angle was 30 to view a field of 220 mm. The slice thickness was at 1.3 mm and acquisition matrix of 256 × 256*.* The hippocampus was measured including the fimbria but with exclusion of the rostral gray matter. The hippocampal tail was included as seen by its oval shape just medial and caudally to the lateral ventricles (Pruessner et al. 2000). It was carried out at Suez Canal University MRI center.

The volume of the hippocampus was calculated by the summation of measured volumes of both right and left hippocampus. The mean value of hippocampus volume among normal elderly was 5.75 cm^3^ with standard deviations of ± 1.1 cm^3^ (Moretti et al. 2007). The cutoff value was presented using two standard deviation from the mean (3.55 cm^3^). So, the atrophy of hippocampus was considered in elderly with hippocampus volume measuring less than that value.

### Digital EEG examination

Digital EEG was recorded utilizing E-Series EEG/PSG system © Compumedics Limited 2004, Australia. Placing the electrodes was done according to the International 10–20 system, and ear lobe electrodes were used as reference, with a ground electrode on the forehead. Impedance was kept below 10 kohm. The high-frequency filter was 70 Hz, the time constant was 0.3, and the paper speed was 30 mm/s. The investigation was carried out while the patient was recumbent in supine position in semi-dark room. Recording was carried out for about 20 min with 3-min hyperventilation and intermittent photic stimulation as provocative techniques.

The EEG records were visually assessed. Five artifacts’ free epochs while awake and resting were selected for QEEG, each epoch of 10-s duration. The relative powers of 19 electrodes (Fp1, Fp2, F7, F8, F3, F4, C3,C4, T3, T4, T5, T6, P3, P4, O1, O2, Fz, Cz, and Pz) were studied in the following frequency bands: delta (0.5 to < 4 Hz), theta (4 to < 8 Hz), alpha 1 (8 to < 11.0 Hz), alpha 2 (11 to < 14 Hz), beta 1 (14 to < 25 Hz), and beta 2 (25 to 35 Hz). The relative power was the percentage of the power of a given frequency band compared to the sum of the power of all frequency bands (Sharbrough et al. 2002).

### Statistical analysis

Collected data were processed using SPSS IBM SPSS statistics (version 22.0, 2013; IBM Corp. Armonk, New York, USA). Quantitative data were presented as means ± SD. On the other hand, qualitative data were presented as numbers and its percentages. The used statistical tests for significance were the following: the one-way ANOVA to test the differences between groups and the chi-squared test in testing significance of difference between qualitative data. Pearson correlation coefficient (*r*) was used to measure correlation between quantitative variables. A probability value (*P* value) of less than 0.05 was considered as statistically significant. A probability value (*P* value) of less than 0.01 was considered statistically highly significant.

## Results

### Demographic data

This study was carried out on 48 subjects divided into three groups, each one containing 16 subjects. The first group consisted of diabetic patients with mild cognitive impairment, and their age ranged between 55 and 80 years with a mean (63.75 ± 6.83 SD); there were seven males (43.8%) and nine females (56.3%). The second group consisted of diabetic patients without cognitive impairment. Their age ranged between 55 and 73 years with a mean (62.18 ± 5.03 SD); there were eight males (50%) and eight females (50%). The third group was the control group; their age ranged between 55 and 71 years with a mean (62.25 ± 4.87 SD). No statistically significant difference was detected between groups as regards age and sex (*P* > 0.05).

### Results of cognitive assessment

On comparing the results of MMSE of the three groups, statistically significant lower results were found in DM with MCI group when compared to other groups (*P* = 0.001) (Table 1).

**Table 1:** The results of cognitive assessment among the study groups

	DM with MCI	DM without MCI	Control group	ANOVA test
	Mean ± SD	Mean ± SD	Mean ± SD	*P* value
	Min–Max	Min–Max	Min–Max	
MMSE	25.5 ± 0.51	28.56 ± 0.81	28.62 ± 0.88)	0.001**
	(25–26)	(28–30)	(28–30)	

*DM* diabetes mellitus, *MCI* mild cognitive impairment, *SD* standard deviation, *Min* minimum, *Max* maximum, *MMSE* Mini Mental State Examination

**Statistically highly significant

### Results of brain imaging

Hippocampal atrophy was distributed significantly among DM with MCI group in comparison to other groups (*P* = 0.01) (Table 2).

**Table 2:** The distribution of hippocampal atrophy among the study groups

	DM with MCI	DM without MCI	Control group	Chi-squared test
				*P* value
Hippocampal atrophy	6 (37.5%)	2 (12.5%)	0 (0.0%)	0.01**

*DM* diabetes mellitus, *MCI* mild cognitive impairment

**Statistically highly significant

### Results of QEEG

Predominance of alpha 2 was significantly higher among DM with MCI group in comparison to other groups with a mean 30.8 ± 12.24 (*P* = 0.01), and alpha 1 was significantly lower among DM with MCI group in comparison to other groups with a mean 20.42 ± 11.87 (*P* = 0.001) while theta, delta, and beta waves was found to be insignificant (Table 3).

**Table 3:** The relative power of all frequency bands among the study groups

	DM with MCI	DM without MCI	Control group	ANOVA test
	Mean ± SD	Mean ± SD	Mean ± SD	*P* value
Delta	3.53 ± 0.57	3.42 ± 0.41	3.68 ± 0.68	0.40
Theta	22.87 ± 3.04	21.54 ± 3.77	21.26 ± 3.85	0.40
Alpha 1	20.44 ± 11.87	31.06 ± 12.70	37.68 ± 5.58	0.001**
Alpha 2	30.80 ± 12.24	20.42 ± 11.78	17.18 ± 4.40	0.01**
Beta	25.75 ± 1.46	24.53 ± 0.68	24.17 ± 1.95	0.33

*DM* diabetes mellitus, *MCI* mild cognitive impairment, *SD* standard deviation

**Statistically highly significant

**Fig. 1:**
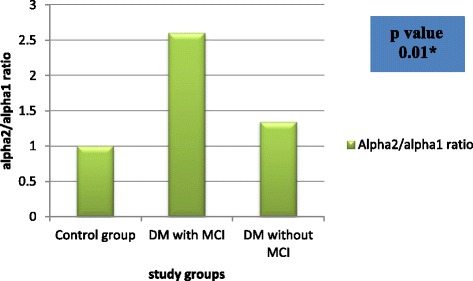
Distribution of alpha 2/alpha 1 power ratio among the study groups. DM = diabetes mellitus, MCI = mild cognitive impairment

Alpha 2/alpha 1 power ratio was distributed significantly among DM with MCI group in comparison to other groups (*P* = 0.01) (Table 4) (Fig. 1).

**Table 4:** The distribution of alpha 2/alpha 1 ratio among the study groups

	DM with MCI	DM without MCI	Control group	ANOVA test
				*P* value
Alpha 2/alpha 1 ratio	2.6 ± 2.15	1.34 ± 1.52	1.0 ± 0.86	0.01**

Alpha 2/alpha 1 power ratio was expressed as mean ± SD

*DM* diabetes mellitus, *MCI* mild cognitive impairment

**Statistically highly significant

**Fig. 2:**
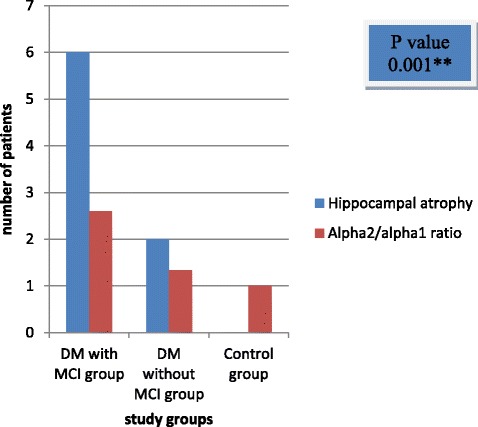
Distribution of hippocampal atrophy and alpha 2/alpha 1 ratio among the study groups. DM = diabetes mellitus, MCI = mild cognitive impairment

Significant relationship was found between alpha 2/alpha 1 ratio and hippocampal atrophy among DM with MCI group in comparison to other groups (*P* < 0.001) (Fig. 2).

There was a high statistically significant positive correlation between hippocampal atrophy and alpha 2/alpha 1 ratio among DM with MCI group, where correlation coefficient (*r*) = 0.535 and *P* = 0.001 respectively (Table 5).

**Table 5:** Correlation between alpha 2/alpha 1 ratio and hippocampal atrophy among DM with MCI group

	Alpha 2/alpha 1 ratio	
	Correlation coefficient (*r*)	*P* value
Hippocampal atrophy	0.535*	0.001**

*DM* diabetes mellitus, *MCI* mild cognitive impairment

*Significant *r* (correlation coefficiency) > 0.5

**Correlation is highly significant at < 0.01

## Discussion

Previous studies support the view that individuals with diabetes are at an increased risk for developing cognitive impairment (Allen et al. 2004) and dementia (Biessels et al. 2006). These studies found indicators for the associations between MCI and type 2 diabetes mellitus. Moreover, another longitudinal cohort study emphasized the association of type 2 diabetes mellitus with so called amnestic MCI among the population who developed both AD and vascular pathology, but with weak association with nonamnestic MCI (Biessels et al. 2007). EEG is not a new idea to be used as an early detection for cognitive decline. But still, EEG is unable to meet the National Institute on Aging Consensus Conference (1998) ideal parameters to be considered as a reliable biomarker. On the other hand, QEEG stood to debate those parameters with the facts of being a cost-effective, noninvasive technique for the identification of the earliest signs of brain dysfunction in patients with evolving dementia or even MCI. That will give the opportunity for early intervention and better chance for therapy to these patients (Prichep 2007). Another study adopted the same concept of considering electroencephalogram as an ideal noninvasive and economic procedure (Rossini et al. 2006).

The current study showed predominance of hippocampal atrophy among DM with MCI group with significant statistical difference (*P* < 0.05), while it was 12.5% of diabetic patient without MCI and 0.0% in normal control group. That was supported by other studies (Moretti et al. 2011; Frisoni 2012; Albert et al. 2011; McKhann et al. 2011).

In the current study, peak power frequency of alpha 2 was found high with predominance with hippocampal atrophy among DM with MCI group. That was supported by other studies (Moretti et al. 2011; Frisoni 2012). O’Donnell and Grace (1995) explained that predominance of synchronized alpha 2 bands over the frontal, temporal, and parietal regions is due to a progressive recruitment of many other cortical areas that involve wider cortico-thalamic re-entry loops. They specified the loop of the frontal-midline thalamic nuclei**.** This explanation was confirmed by Nicolelis and Fanselow (2002) and Klimesch et al. (2007) who considered the increase in the alpha power to be due to hyperpolarization at thalamic level.

The current study found significant predominance of alpha 1(low alpha) among subjects without cognitive impairment, and that was in agreement with that reported by Jelic et al. (2000) and considered these changes as a predictor of future conversion from MCI to AD. Moreover, Klimesch et al. (2003) stated that induction of large alpha 1 power by neurofeedback training or repetitive transcranial magnetic stimulation (rTMS) at alpha frequency range is typical for good memory performance under normal situations enhancing the cognitive performance.

That also was confirmed by Holschneider et al. (1998) and Sarter and Bruno (1997, 1999, 2000) who reported that the efferent projections of the forebrain are the main providers of the low alpha power. They regarded that to the cholinergic tone of the base of cortex especially in the nucleus basalis of Meyner. But some studies actually considered that alpha 1 is related to hippocampal atrophy (Rossini et al. 2006). In the current study, alpha 2/alpha 1 power ratio was found significant among patient with hippocampal atrophy and this was supported by Moretti et al. (2011) and Frisoni (2012).

Finally, type 2 DM was suggested by Roberts et al. (2014) to be the factor of increasing the risk of cognitive impairment acceleration and development of MCI into dementia, furthermore resulting in hippocampal atrophy which may end in QEEG changes (Ganguli et al. 2004; Hussain 2007; Xu et al. 2010). But unfortunately, there were no available data about the effect of DM on QEEG. So the current study points out that DM could directly result in QEEG changes as well as resulting in hippocampal atrophy which may open the door to enrich the idea for further studies to consider using QEEG as an alternative predictor of hippocampal atrophy for mild cognitive impairment.

## Conclusions

Type 2 DM was suggested to increase the risk of cognitive impairment. The cognitive impairment in patients with diabetes mellitus was associated with changes in hippocampal volume and QEEG changes. Follow-up assessment of cognitive function of diabetic patients is recommended to detect early cognitive dysfunction, and proper glycemic control may have a good prognostic value in cognitive performance in diabetic patients.
